# Timing of readmissions for complications following emergency colectomy: follow-up beyond post-operative day 30 matters

**DOI:** 10.1007/s00464-024-10724-y

**Published:** 2024-03-19

**Authors:** Natasha G. Caminsky, Jeongyoon Moon, Daniel Marinescu, Allison J. Pang, Carol-Ann Vasilevsky, Marylise Boutros

**Affiliations:** 1 Division of Colon and Rectal Surgery, Sir Mortimer B. Davis Jewish General Hospital, Montreal, QC Canada; 2 Department of Colorectal Surgery, Cleveland Clinic Florida (Weston Hospital), Weston, FL USA

**Keywords:** Emergency colectomy, Late readmission, Complications

## Abstract

**Background and purpose:**

Emergency colectomies are associated with a higher risk of complications compared to elective ones. A critical assessment of complications occurring beyond post-operative day 30 (POD30) is lacking. This study aimed to assess the readmission rate and factors associated with readmission 6-months following emergency colectomy.

**Methods:**

A retrospective cohort study of adult patients who underwent emergency colectomy (2010–2018) was performed using the Nationwide Readmissions Database. The cohort was divided into two groups: (i) no readmission and (ii) emergency readmission(s) for complications related to colectomy (defined using ICD-9/10 codes). Readmissions were categorized as either “early” (POD0–30) or “late” (> POD30). Differences between groups were described and multivariable regression controlling for relevant covariates defined a priori were used to identify factors associated with timing of readmission and cost.

**Results:**

Of 141,481 eligible cases, 13.22% (*n* = 18,699) were readmitted within 6-months of emergency colectomy for colectomy-related complications, 61.63% of which were “late” readmissions (> POD30). The most common reasons for “late” readmission were for bleeding, gastrointestinal, and infectious complications (20.80%, 25.30%, and 32.75%, respectively). On multiple logistic regression, female gender (OR 1.12; 95%CI 1.04–1.21), open procedures (OR 1.12, 95%CI 1.011–1.24), and sigmoidectomies (OR 1.51, 95%CI 1.39–1.65, relative to right hemicolectomies) were the strongest predictors of “late” readmission. On multiple linear regression, “late” readmissions were associated with a $1717.09 USD (95%CI $1717.05–$1717.12) increased cost compared to “early” readmissions.

**Discussion:**

The majority of colectomy-related readmissions following emergency colectomy occur beyond POD30 and are associated with cases that are of overall higher morbidity, as well as open sigmoidectomies. Given the associated increased cost of care, mitigation of such readmissions by close follow-up prior to and beyond POD30 is advisable.

**Supplementary Information:**

The online version contains supplementary material available at 10.1007/s00464-024-10724-y.

The complication rate following emergency colorectal surgery is almost twice that following an elective procedure [1, 2]. When characterizing post-operative “late” complications following colorectal surgery, studies limit follow-up to post-operative day 30 (POD30), except for recurrence and mortality, which are often reported up to 5-years post-operatively. While some of these post-operative complications can be treated on an out-patient basis, many require readmission and even reoperation. A retrospective review of 69,222 elective colorectal procedures demonstrated that 10.8% of patients required readmission within the 30-day post-operative period and that readmissions occurring beyond POD5 were associated with patients who had metastatic disease, had creation of a stoma, and had post-operative urinary tract complications [3]. In a retrospective cohort study of 1736 colon and rectal resections performed at a single institution from 2014 to 2018, 11% had an emergency department visit within 30 days of discharge. One third of these visits were deemed preventable and 69% of these emergency department visits led to readmission [4].

To our knowledge, a critical assessment and characterization of complications occurring beyond POD30 following emergency colorectal procedures has yet to be performed. The objective of this study was to assess readmission rates and factors associated with readmission in the 6-months following emergency colectomy surgery using the Nationwide Readmissions Database (NRD). We hypothesized that an important proportion of complications requiring readmission following emergency colectomy occurs beyond the POD30 cut-off.

## Methods

This study received institutional review board exemption and is reported following the STrengthening and Reporting of Observational studies in Epidemiology (STROBE) guidelines [5] Online Appendix 1.

### Data source

This was a retrospective cohort study using the NRD, which was developed by the Agency for Healthcare Research and Quality (AHRQ) as part of the Healthcare Cost Utilization Project (HCUP). Representing data from all states, NRD captures and links all admissions for a given patient within the calendar year, making it possible to identify readmissions following surgery. NRD is one of the largest available discharge databases and approximately 18 million discharges are recorded each year and all payers, including the uninsured, are captured, making this database strongly representative. Variables provided by the database include demographics, comorbidities, admission information [(diagnosis, procedures, length of stay (LOS)], cost of stay, type of hospital, insurance type, and household income.

### Patient population

NRD was queried from 2010 to 2018 to identify all adult (age ≥ 18 years) patients who underwent colectomy (see Online Appendix 2 for ICD-9/10 procedure codes—cases where only a stoma was created were not included). Given that patients cannot be linked from 1 calendar year to the next in NRD, only patients operated on between January 1st and June 30th of each year were retained to allow for a 6-month follow-up for all patients.

Cases were excluded from the study if the patient died during admission or if the admission was coded as being either elective or did not have clear coding (i.e., invalid or missing coding for admission type). Thus, only cases of emergency colectomy remained.

**Fig. 1:**
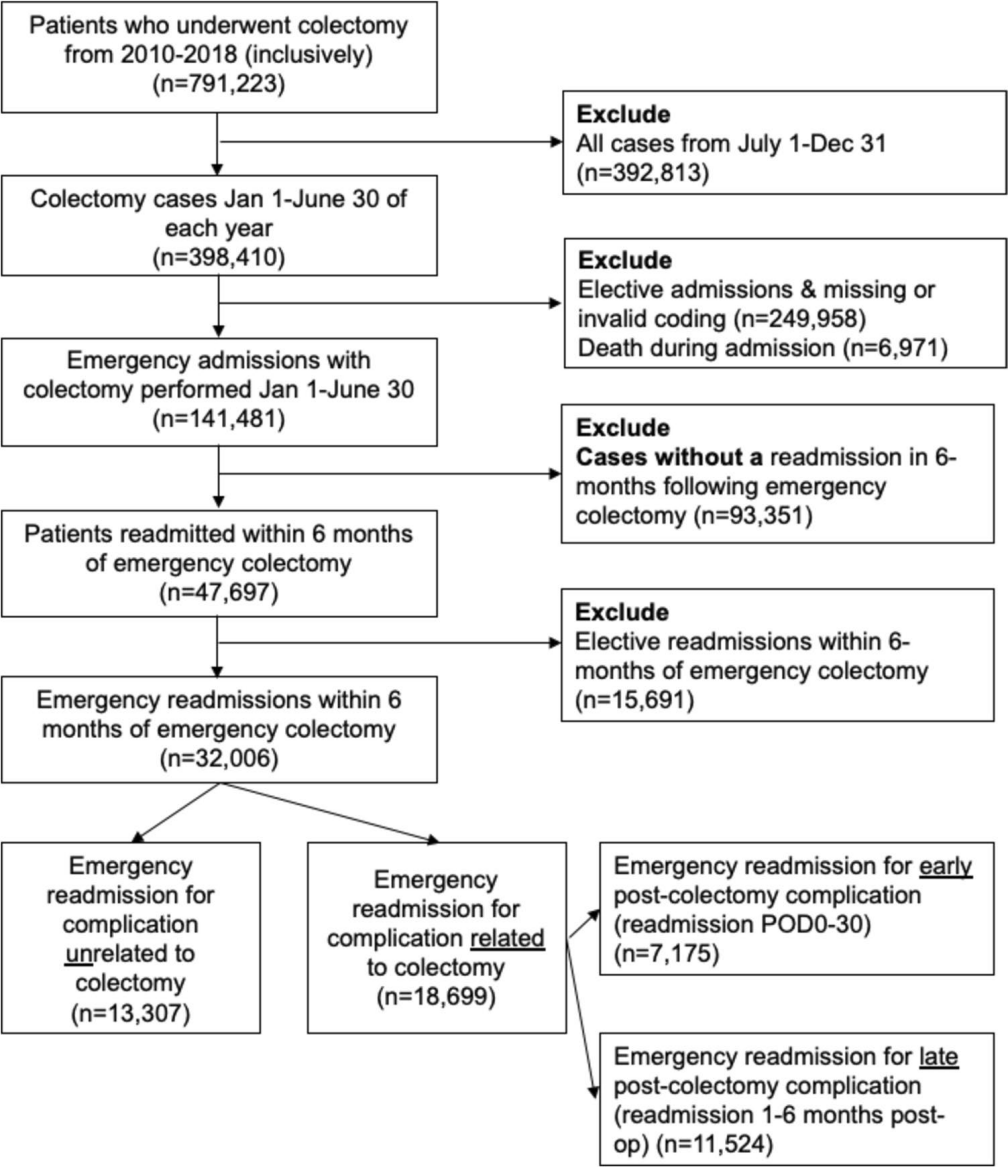
Patient flow diagram

Readmissions for these cases of emergency colectomy were then identified. Cohort entry was defined as the date of emergency colectomy and cohort exit was defined as 6-months post-colectomy. Patients without a readmission were excluded and readmissions that were labeled as elective were also excluded (Fig. 1).

### Covariates

Data were collected on patient demographics (age, sex); individual comorbidities; All Patient Refined-Diagnosis Related Group (APR-DRG) Severity of Illness Score; APR-DRG Risk of Mortality Score; surgical approach (minimally invasive, conversion to open, and open); type of colectomy [right hemicolectomy (which includes ileocecectomy), transverse colectomy, left hemicolectomy, segmental colectomy, sigmoidectomy, or total colectomy], LOS for admission following emergency colectomy; history of chemotherapy; history of radiotherapy; malignant vs benign disease; teaching status of the hospital; hospital size; median household income; and patient’s urban vs rural location (dichotomized as ≥ 1 million population vs < 1 million population). Finally, we created a variable *origin vs hospital size concordance* to indicate whether patients were treated in a hospital close to where they reside (i.e., did the patient have to travel outside of their region to receive care). To accomplish this, the variable *population served by hospital* was dichotomized into *large* (“Large metropolitan areas with ≥ 1 million residents”) vs *small* (all other categories) and the variable *area of origin* was dichotomized into *large* (“Central” counties of metro areas of ≥ 1 million population or “Fringe” counties of metro areas of ≥ 1 million population) and *small* (all other categories). For each case, if both of these new categories had the same designation (i.e., *large* and *large* or *small* and *small*), then the variable *origin vs hospital size concordance* was assigned the value *Yes*. If the variables did not have the same designation, then the variable was assigned the value *No*.

### Defining colectomy-related complications

Emergency readmissions that occurred in the 6-months following emergency colectomy were categorized into “readmissions for colectomy-related complications” and “readmissions unrelated to colectomy.” To do this, the first two diagnostic codes and the first two procedure codes for each readmission were screened using a list of ICD9/10 diagnostic and procedure codes deemed to be “colectomy-related complications”. This list of ICD-9/10 diagnostic and procedure codes was previously used to define post-operative colorectal complications [6, 7]. In addition, through an iterative process, the 200 most frequent diagnosis and procedure codes for the “non-colectomy related complication” group were reviewed to ensure that no complications that could be colectomy-related were missed. Any relevant diagnosis or procedure codes were added to the list of “colectomy-related complications” and the sub-groups were re-created using the new list of diagnosis and procedure codes. The process of creating sub-groups of readmissions for “colectomy-related” and “non-colectomy related” complications was repeated until no colectomy-related diagnoses/procedures were left within the “non-colectomy related” sub-group with a frequency > 50. The complete list of diagnosis and procedure codes used in the final analysis is listed in Online Appendix 3.

### Outcomes

The primary outcome of interest was timing of emergency readmission for colectomy-related complications. Timing of emergency readmission was defined as “early” (readmission on POD0–30 post-emergency colectomy) or “late” (readmission between POD31 and 6-months following emergency colectomy). See Fig. 1 for a visual representation of the sub-division of the cohort.

Secondary outcomes of interest included reason for readmission (diagnosis/procedure), cost of readmission (USD), and LOS during readmission (days).

### Subgroup analysis

In order to compare risk factors for readmission for complications following different types of colectomy, the cohort was divided into “right-sided colectomy” (ICD codes for “right hemicolectomy”) and “left-sided colectomy” (ICD codes for “left hemicolectomy,” “segmental resection,” and “sigmoidectomy”).

### Statistical analysis

Data were reported as frequencies with proportions, means with standard deviations, or medians with interquartile ranges (Q1–Q3), as appropriate. Means were compared using a 2-tailed Student *t* test for normally distributed values or the Wilcoxon rank-sum test for non-parametric data. The *χ*^2^ test was used to compare the distribution of categorical variables. Missing data were excluded from univariate analysis. Multiple logistic regression was used to identify predictors of readmission for stoma-related complications (compared to no complications and non-stoma-related complications). Covariates for the model were selected based on previous subject matter knowledge (clinical significance) and statistical significance from the univariate analysis (*p* < 0.05). Finally, multivariable regression analysis was used to identify clinical risk factors for “late” readmission for colectomy-related complications and cost of readmission. Cases with missing data for the variables selected for regression analysis were excluded. Data were analyzed using SAS EG 7.1.

## Results

Of the 791,223 patients who underwent colectomy from 2010 to 2018, 398,410 (50.35%) were admitted in the first half of the calendar year. After excluding elective cases (249,958; 62.73%) and patients who died during the admission (6971; 1.75%), the study cohort consisted of 141,481 cases of emergency colectomies (Fig. 1). In the 6-month follow-up period, there were 47,702 readmissions, of which 18,699 (58.42%) were emergency readmissions for colectomy-related complications (Table 1).

**Table 1:** Cohort demographics

Variable*	Cohort (*n* = 18,699)	“Early” readmissions for colectomy-related complications (*n* = 7175)	“Late” readmissions for colectomy-related complications (*n* = 11,524)	*p* value	Missing
Patient characteristics					
Age, years	67 (54–77)	65 (52–76)	67 (55–77)	< 0.0001	0
Sex				< .00001	0
Female	10,004 (53.50%)	3709 (51.69%)	6295 (54.63%)		
Male	8695 (46.50%)	3466 (48.31%)	5229 (45.37%)		
Patient location: National Center for Health Statistics (NCHS) urban–rural code				0.0158	4862
“Central” counties of metro areas of ≥ 1 million population	3789 (27.39%)	1355 (25.89%)	2434 (28.30%)		
“Fringe” counties of metro areas of ≥ 1 million population	3675 (26.56%)	1377 (26.30%)	2298 (26.71%)		
Counties in metro areas of 250,000–999,999 population	3116 (22.52%)	1205 (23.01%)	1911 (22.21%)		
Counties in metro areas of 50,000–249,999 population	1291 (9.33%)	509 (9.72%)	782 (9.10%)		
Micropolitan counties	1130 (8.16%)	457 (8.73%)	673 (7.82%)		
Not metropolitan or micropolitan counties	836 (6.04%)	332 (6.35%)	504 (5.85%)		
Income quartile				0.7003	285
1	5064 (27.50%)	1972 (27.94%)	3092 (27.22%)		
2	4684 (25.44%)	1792 (25.40%)	2892 (25.47%)		
3	4722 (25.64%)	1784 (25.28%)	2938 (25.87%)		
4	3944 (21.42%)	1509 (21.38%)	2435 (21.44%)		
All Patient Refined—Diagnosis Related Group (APR-DRG): severity of illness subclass				< 0.0001	2
Minor loss of function**	1691 (9.04%)	908 (12.66%)	783 (6.79%)		
Moderate loss of function	5400 (28.88%)	2402 (33.48%)	33 (26.02%)		
Major loss of function	6996 (37.41%)	2586 (36.04%)	36 (38.27%)		
Extreme loss of function	4610 (24.65%)	1279 (17.83%)	18 (28.90%)		
APR-DRG: risk of mortality subclass				< 0.0001	2
Minor likelihood of dying	4526 (24.20%)	2244 (31.28%)	2282 (19.80%)		
Moderate likelihood of dying	4683 (25.04%)	1847 (25.74%)	2836 (24.61%)		
Major likelihood of dying	5843 (31.25%)	2054 (28.63%)	3789 (32.88%)		
Extreme likelihood of dying	3645 (19.49%)	1030 (14.36%)	2615 (22.69%)		
Comorbidities					
Acquired immune deficiency syndrome (AIDS)	20 (0.17%)	0 (0.00%)	15 (0.20%)	0.2074	6654
Alcohol abuse	349 (2.90%)	113 (2.42%)	236 (3.20%)	0.0136	6654
Deficiency anemias	3,111 (25.83%)	1,055 (22.62%)	2,056 (27.85%)	< 0.0001	6654
Rheumatoid arthritis/collagen vascular diseases	417 (3.46%)	138 (2.95%)	279 (3.78%)	0.0163	6654
Chronic blood loss anemia	463 (3.85%)	160 (3.43%)	303 (4.11%)	0.0607	6654
Congestive heart failure	1177 (9.77%)	385 (8.26%)	792 (10.73%)	< 0.0001	6654
Chronic pulmonary disease	2243 (18.63%)	836 (17.92%)	1407 (19.06%)	0.1181	6654
Coagulopathy	652 (5.42%)	209 (4.48%)	443 (6.00%)	0.0003	6654
Depression	1184 (9.83%)	429 (9.20%)	755 (10.23%)	0.0642	6654
Diabetes, uncomplicated	2129 (17.68%)	815 (17.48%)	1314 (17.80%)	0.6455	6654
Diabetes with chronic complications	378 (3.14%)	132 (2.83%)	246 (3.33%)	0.1232	6654
Drug abuse	276 (2.30%)	100 (2.14%)	176 (2.39%)	0.3904	6654
Hypertension (both complicated and uncomplicated)	6669 (55.37%)	2536 (54.37%)	4133 (56.00%)	0.0813	6654
Hypothyroidism	1438 (11.94%)	555 (11.91%)	883 (11.96%)	0.9166	6654
Liver disease	308 (2.56%)	92 (1.97%)	216 (2.92%)	0.0012	6654
Lymphoma	132 (1.10%)	42 (0.91%)	90 (1.22%)	0.1016	6654
Fluid and electrolyte disorders	5042 (41.86%)	1748 (37.48%)	3294 (44.62%)	< 0.0001	6654
Metastatic cancer	1665 (13.82%)	560 (12.00%)	1105 (14.97%)	< 0.0001	6654
Other neurologic disorders	809 (6.72%)	289 (6.20%)	520 (7.04%)	0.0699	6654
Obesity	1506 (12.50%)	541 (11.60%)	965 (13.07%)	0.0172	6654
Paralysis	332 (2.76%)	98 (2.11%)	234 (3.17%)	0.0005	6654
Peripheral vascular disorders	1021 (8.48%)	353 (7.57%)	353 (7.57%)	0.0045	6654
Psychoses	535 (4.44%)	198 (4.25%)	337 (4.56%)	0.4056	6654
Pulmonary circulation disorders	374 (3.10%)	116 (2.49%)	258 (3.50%)	0.0019	6654
Renal failure	1292 (10.73%)	413 (8.86%)	879 (11.91%)	< 0.0001	6654
Solid tumor without metastasis	492 (4.08%)	166 (3.55%)	326 (4.42%)	0.0205	6654
Peptic ulcer disease excluding bleeding	0 (0.00%)	0 (0.00%)	0 (0.00%)	0.0245	6654
Valvular disease	588 (4.88%)	197 (4.23%)	391 (5.29%)	0.0077	6654
Weight loss	2011 (16.69%)	590 (12.65%)	1421 (19.25%)	< 0.0001	6654
Disease characteristics					
Benign disease	18,345 (98.11%)	7053 (98.30%)	11,292 (97.99%)	0.126	0
History of chemotherapy	313 (1.67%)	105 (1.46%)	208 (1.80%)	0.0767	0
History of radiotherapy	312 (1.67%)	117 (1.63%)	195 (1.69%)	0.7497	0
Colectomy characteristics					
Approach				< 0.0001	0
MIS***	3020 (16.15%)	1286 (17.92%)	1734 (15.05%)		
Open****	15,679 (83.85%)	5889 (82.08%)	9790 (84.95%)		
Resection type				< 0.0001	0
Right hemicolectomy	8095 (43.29%)	3438 (47.92%)	4657 (40.41%)		
Transverse colectomy	64 (3.55%)	238 (3.32%)	426 (3.70%)		
Left hemicolectomy	1866 (9.98%)	655 (9.13%)	1211 (10.51%)		
Sigmoidectomy	5965 (31.90%)	2152 (29.99%)	3813 (33.09%)		
Segmental resection	1691 (9.04%)	553 (7.71%)	1138 (9.88%)		
Total colectomy	418 (2.24%)	139 (1.94%)	279 (2.42%)		
Index admission characteristics					
LOS, days	10 (7–15)	8 (6–12)	11 (7–18)	< 0.0001	0
Total charge, USD	93,917.50 (57,671–161,099)	80,697 (51,587–128,826)	105,623 (62,516–185,457)	< 0.0001	0
Disposition of patient (following emergency colectomy)				< 0.0001	14
Routine	7397 (39.59%)	3498 (48.78%)	3899 (33.86%)		
Transfer to short-term hospital	158 (0.84%)	44 (0.61%)	114 (0.99%)		
Transfer other*****	5648 (30.22%)	1743 (24.3%)	3905 (33.92%)		
Home Health Care (HHC)	5410 (28.95%)	1843 (25.71%)	3567 (30.98%)		
Against Medical Advice (AMA)	72 (0.39%)	43 (0.60%)	29 (0.25%)		
Teaching status of hospital				0.1136	0
Metropolitan non-teaching	6753 (36.11%)	2633 (36.70%)	4120 (35.75%)		
Metropolitan teaching	10,559 (56.47%)	3987 (55.56%)	6572 (57.03%)		
Non-metropolitan	1387 (7.42%)	555 (7.74%)	832 (7.22%)		
Hospital size				0.2387	0
Small	2455 (13.13%)	980 (13.66%)	1475 (12.80%)		
Medium	5258 (28.12%)	2007 (27.97%)	3251 (28.21%)		
Large	10,986 (58.75%)	4188 (58.37%)	6798 (58.99%)		
Hospital urban–rural designation				0.0074	0
Large metropolitan areas ≥ 1 million residents	10,757 (57.53%)	4014 (55.94%)	6743 (58.51%)		
Small metropolitan areas with < 1 million residents	6555 (35.06%)	2606 (36.32%)	3949 (34.27%)		
Micropolitan areas	1130 (6.04%)	451 (6.29%)	679 (5.89%)		
Not metropolitan or micropolitan (non-urban residual)	257 (1.37%)	104 (1.45%)	153 (1.33%)		
Origin vs hospital size concordance, *Yes*	13,084 (94.55%)	4928 (94.13%)	8156 (94.82%)	0.0875	4862

*Values are expressed as median (Q1–Q3) for continuous variables and *n* (%) for categorical variables unless otherwise specified; **Includes cases with no comorbidity or complications; ***MIS (minimally invasive) includes laparoscopic and robotic; ****Includes open and MIS-converted to open; *****Includes skilled nursing facility (SNF), intermediate care facility (ICF), another type of facility

### Primary outcome

**Fig. 2:**
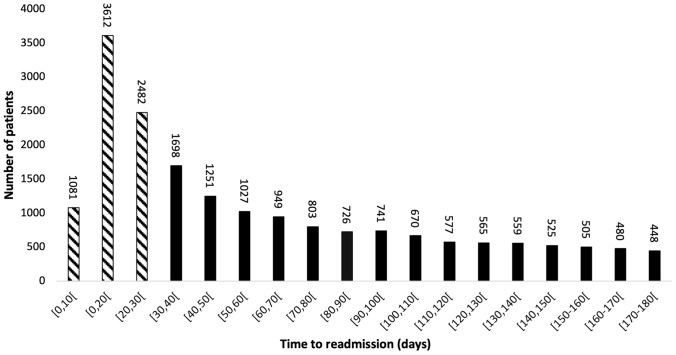
Distribution of time to readmission for patients with emergency readmissions for colectomy-related complications. Arrow indicates median time to readmission for cohort (43 days)

The median time to readmission for colectomy-related complications was 43 days (IQR 19–95) (Fig. 2). The proportion of colectomy-related complications requiring “early” readmission (i.e., ≤ POD30) was 38.37% [*n* = 7175, median time to “early” readmission 16 days (IQR 11–22)], while the proportion of “late” readmissions for colectomy-related complications was 61.63% [*n* = 11,524, median time to readmission 80 days (IQR 49–123)].

### Secondary outcomes

Renal and stoma-related complications were significantly more prevalent in the “late” readmission group (19.60% and 12.18%, respectively) compared to the “early” readmission group (12.23% and 2.45%, respectively; *p* < 0.001). The most common complications leading to readmission in both groups were bleeding, gastrointestinal, and infectious complications (Table 2). Significantly more patients in the “early” readmission group required interventions related to a post-operative complication compared to the “late” group (18.17% vs 12.14%, *p* < 0.0001). The interventions that patients in the “late” readmission group underwent during readmission were percutaneous drainages (*n* = 699), re-laparotomy and lavage (*n* = 491), incision and drainage (*n* = 192), and incisional hernia repair (*n* = 17). Bleeding complications resulting in readmission were associated with the administration of blood products 75% of the time. There was no difference in LOS for the readmission [5 days (IQR 3–9) for both; *p* = 0.4928] between the “early” and “late” groups, nor was there a statistically significant difference in the cost of readmission [34,374 USD (IQR 18,386–68,014) vs 37,936 USD (IQR 20,464–73,835), *p* = 0.0532].

**Table 2:** Outcomes

Variables	Cohort (18,699)	“Early” readmissions for colectomy-related complications (*n* = 7175)	“Late” readmissions for colectomy-related complications (*n* = 11,524)	*p* value
LOS, days	5 (3–9)	5 (3–9)	5 (3–9)	0.4928
Total charge, USD	36,566 (19,576–71,578)	34,374 (18,386–68,014)	37,936 (20,464–73,835)	0.0532
Time to readmission, days from colectomy	43 (19–95)	16 (11–22)	80 (49–123)	< 0.0001
Reason for readmission*^a^				< 0.0001
Anastomotic leak	2006 (7.62%)	1270 (17.70%)	736 (6.39%)	
Bleeding	4029 (15.30%)	1631 (22.73%)	2397 (20.80%)	
Failure to thrive	99 (0.38%)	27 (0.38%)	72 (0.62%)	
Gastrointestinal	4638 (17.62%)	1722 (24.00%)	2916 (25.30%)	
Infection	6656 (25.28%)	2882 (40.17%)	3774 (32.75%)	
Intervention for post-operative complication	2703 (10.27%)	1304 (18.17%)	1399 (12.14%)	
Post-operative pain	141 (0.54%)	87 (1.21%)	54 (0.47%)	
Renal	3208 (12.19%)	949 (13.23%)	2259 (19.60%)	
Stoma-related complication	1580 (6.00%)	176 (2.45%)	1404 (12.18%)	
Venous thromboembolism (including pulmonary embolism)	671 (2.55%)	251 (3.50%)	420 (3.64%)	
Ventral/incisional hernia	102 (0.39%)	16 (0.22%)	86 (0.75%)	
Wound complication	495 (1.88%)	268 (3.74%)	227 (1.97%)	

Values are expressed as median (Q1–Q3) for continuous variables and *n* (%) for categorical variables unless otherwise specified

*Please see Online Appendix 3 for the list of specific complications that fall into each of these categories

^a^The sum of each column adds up to > 100%, as more than one reason for readmission can be attached to each individual case

### Univariate analysis

Patients with “late” readmissions for colectomy-related complications were more likely to have deficiency anemias (27.85% vs 22.62%, *p* < 0.0001), electrolyte disturbances (44.62% vs 37.48%, *p* < 0.0001), renal failure (11.91% vs 8.86%, *p* < 0.0001), and a history of weight loss (19.25% vs 12.65%, *p* < 0.0001). These patients were also more often at risk for extreme loss of function following emergency colectomy (28.9% vs 17.83%, *p* < 0.0001) and had extreme likelihood of dying (22.69% vs 14.36%, *p* < 0.0001) as per the APR-DRG mortality risk score, were much less likely to have a “routine” discharge (33.86% vs 48.78%, *p* < 0.0001), and had a longer LOS for the emergency colectomy [11 (IQR 7–18) days vs 8 (IQR 6–12) days, *p* < 0.0001]. The most common procedure responsible for patients who had “early” readmission for colectomy-related complications following emergency colectomy was right hemicolectomy (47.92% vs 40.41%, *p* < 0.0001), while “late” readmission for colectomy-related complications was more common following sigmoidectomy (33.09% vs 29.99%, *p* < 0.0001) (Table 1).

### Multivariable regression analysis

#### Factors associated with “late” readmission for colectomy-related complications

The following covariates were selected for multivariable regression analysis for “late” readmission: age, gender, surgical approach, weight loss, electrolyte disturbances, renal failure, congestive heart failure, deficiency anemia, APR-DRG risk of mortality subclass, and type of colectomy. Female patients had a 12% (95%CI 1.04–1.21) greater odds of “late” readmission compared to males and those who underwent open emergency colectomy had a 12% greater odds of “late” emergency readmission compared to a laparoscopic approach (95%CI 1.01–1.24). A history of recent weight loss was also a strong predictor of “late” emergency readmission with 32% greater odds compared to those without a history of recent weight loss. When compared to patients with a low predicted likelihood of dying as per APR-DRG following emergency colectomy, patients with an extreme predicted risk of dying had 111% greater odds of “late” readmission (95%CI 1.83–2.43). Finally, emergency sigmoidectomy was associated with the highest odds of “late” readmission when compared to right hemicolectomy (OR 1.51, 95%CI 1.39–1.65) (Table 3).

**Table 3:** Multivariable logistic regression for odds of “early” vs “late” readmission for colectomy-related complications following emergency colectomy

Co-variate	OR	95% CI
Age	1.00	1.00–1.00
Female	1.12	1.04–1.21
Laparoscopic approach	0.89	0.81–0.99
Weight loss	1.32	1.18–1.48
Electrolyte disturbances	1.052	0.97–1.14
Renal failure	1.11	0.98–1.27
Congestive heart failure	1.02	0.89–1.17
Deficiency anemia	1.19	1.09–1.30
Moderate likelihood of dying*	1.49	1.34–1.65
Major likelihood of dying*	1.72	1.54–1.93
Extreme likelihood of dying*	2.11	1.83–2.43
Transverse colectomy**	1.27	1.04–1.55
Left hemicolectomy**	1.36	1.20–1.54
Sigmoidectomy**	1.51	1.397–1.65
Segmental resection**	1.50	1.28–1.75
Total colectomy**	1.47	1.18–1.82

*Versus *Minor likelihood of dying*; **Versus *right hemicolectomy*

#### Factors associated with cost of readmission for colectomy-related complications

When controlling for relevant covariates (timing of readmission, age, gender, surgical approach, weight loss, electrolyte disturbances, renal failure, congestive heart failure, deficiency anemia, APR-DRG risk of mortality subclass, and type of colectomy), “late” readmissions for colectomy-related complications were associated with a $1717.09 USD (95%CI $1717.05–$1717.12 USD) greater cost compared to “early” readmissions (Table 4). Important factors associated with cost of readmission include male sex, namely $3451.08 USD (95%CI $3451.04–$3451.12 USD), when compared to females. Open cases were associated with a marginal increase in cost of $529.97 USD (95%CI $529.91–$530.02 USD). Conversely, a 1-year increase in age was associated with a decrease of $152.75 USD. Finally, of all the resection types, sigmoidectomies were associated with the greatest increase in cost of readmission when compared to right hemicolectomies [$2929.84 USD (95%CI $2929.782–$2929.90 USD)].

**Table 4:** Multivariable linear regression for predictors of cost of readmission for colectomy-related complications following emergency colectomy

Parameter	OR	95% CI
Intercept	55,543.88	55,543.80 to 55,543.96
Female	− 3451.08	− 3451.12 to − 3451.04
Age	− 152.75	− 152.75 to − 152.75
Laparoscopic approach	− 529.97	− 530.02 to − 529.91
Weight loss	6931.95	6931.90 to 6932.01
Electrolyte disturbance	4408.64	4408.60 to 4408.68
Renal failure	5189.36	5189.30 to 5189.43
Congestive heart failure	6489.94	6489.87 to 6490.00
Deficiency anemia	5991.73	5991.69 to 5991.77
Moderate likelihood of dying*	5830.84	5830.79 to 5830.89
Major likelihood of dying*	12,727.04	12,726.99 to 12,727.10
Extreme likelihood of dying*	24,434.88	24,434.81 to 24,434.94
Transverse colectomy**	2333.96	2333.86 to 2334.06
Left hemicolectomy**	2929.84	2929.782 to 2929.90
Sigmoidectomy**	1097.71	1097.66 to 1097.75
Segmental resection**	1531.05	1530.97 to 1531.12
Total colectomy**	− 3559.80	− 3559.91 to − 3559.70
“Late” readmission (30 days–6 months post-emergency colectomy)***	1717.09	1717.05 to 1717.12

*Versus *Minor likelihood of dying*; **Versus *right hemicolectomy*; ***Versus “early” readmission (≤ 30 days post-emergency colectomy)

### Subgroup analysis

A significantly greater proportion of “late” readmissions occurred for left- vs right-sided resections (64.71% vs 57.53%, *p* < 0.0001) (Table 5). There were more factors predictive of “late” readmission for right-sided resections: female sex (OR 1.266, 95%CI 1.125–1.425), weight loss (OR 1.668, 95% CI 1.403–1.984), electrolyte disturbances (OR 1.153, 95% CI 1.017–1.308), and extreme risk of mortality score (OR 2.082, 95% CI 1.752–2.473) (Table 6). This is compared to left-sided resections, which only had two significant predictors: deficiency anemias (OR 1.159, 95% CI 1.017–1.32) and an extreme risk of mortality score (OR 1.737, 95% CI 1.427–2.114) (Table 7). When comparing right-sided and left-sided resections, the LOS on readmission for colectomy-related complications was not significantly different (*p* = 0.2375). Refer to Online Appendix 4 for univariate analysis comparing right versus left colectomies.

**Table 5:** Outcomes for emergency colectomies (right vs left hemicolectomy)

Variables	Left-sided colectomy (*n* = 9522)	Right-sided colectomy (*n* = 8095)	*p* value
LOS, days	5 (3–8)	5 (3–9)	0.2375
Total charge, USD	35,131 (18,901–67,813)	37,882.5 (20,209–74,799)	0.0452
Time to readmission, days from colectomy	38 (17–86)	48 (21–100)	< 0.0001
Timing of readmission			< 0.0001
“Early”	3360 (35.29%)	3438 (42.47%)	
“Late”	6162 (64.71%)	4657 (57.53%)

**Table 6:** Predictors of “late” readmission for right-sided colectomy sub-group

Variable	OR	95% CI
Age	1.00	1.00–1.00
Female	1.27	1.13–1.43
Laparoscopic approach	0.98	0.85–1.14
Weight loss	1.67	1.40–1.98
Electrolyte disturbances	1.15	1.02–1.31
Renal failure	1.18	0.97–1.43
Congestive heart failure	1.07	0.87–1.31
Deficiency anemia	1.26	1.10–1.44
Moderate likelihood of dying*	2.13	1.80–2.51
Major likelihood of dying*	2.08	1.75–2.47
Extreme likelihood of dying*	2.74	2.19–3.44

*Based on the APR-DRG Risk of Mortality Score

**Table 7:** Predictors of “late” readmission for left-sided colectomy sub-group

Variable	OR	95% CI
Age	1.00	0.99–1.00
Female	1.03	0.93–1.15
Laparoscopic approach	0.83	0.72–0.97
Weight loss	1.06	0.90–1.23
Electrolyte disturbances	0.99	0.88–1.11
Renal failure	1.03	0.85–1.25
Congestive heart failure	1.01	0.83–1.23
Deficiency anemia	1.16	1.02–1.32
Moderate likelihood of dying*	1.18	1.022–1.37
Major likelihood of dying*	1.51	1.30–1.77
Extreme likelihood of dying*	1.74	1.43–2.11

*Based on the APR-DRG Risk of Mortality Score

## Discussion

In this retrospective review of 141,481 emergency colectomies (2010–2018) in adult patients, 13.22% had emergency readmissions within 6-months for colectomy-related complications. The majority (61.63%) of these occurred in what was defined as the “late” readmission period (> 30 days post-colectomy). A previous study of unplanned readmissions in the 30-days post-discharge from both elective and emergency procedures in 9 common surgical specialties found that 8.8% had unplanned readmissions [8], which is substantially lower than our study (where we found a 22.6% emergency readmission rate in the 6-month post-operative period); however this could be due to the fact that < 7% of these cases were colectomies. Also, having a cohort of only emergency cases, along with a longer follow-up period, may further account for the observed difference. Another study, that explicitly explored follow-up after 30 days, looked at just over 10,000 adults (18–65 years) with a BlueCross BlueShield health plan having undergone a colorectal procedure (elective and emergency) and found that 23.3% had a readmission within 90-days after discharge [9]. Readmissions were similarly subdivided into “early” and “late” readmissions; 52.6% occurred in the first 30 days post-discharge and the remainder between 31 and 90 days post-discharge, proportions that are similar to our study, which only looked at emergency colectomy.

In our study, the three main reasons for readmission in both the “early” and “late” readmission periods were infection, complications related to the gastrointestinal tract (including ileus, persistent vomiting, need for total parenteral nutrition, adhesive bowel obstruction), and bleeding. Interestingly, however, compared to our study, the burden of stoma-related complications and renal complications was 4.97 and 1.48 times greater, respectively, in the “late” readmission group compared to the “early” readmission group. Wong et al. identified similar reasons for emergency readmission in a retrospective review of 1763 colectomies and proctectomies and associated emergency readmissions within the 30-day post-operative period, with the most common reasons for readmission being ileus/nausea and vomiting (12%), intra-abdominal abscess or leak (13%) and superficial surgical site infections (8%) [4].

Predictors of “late” readmission included female gender, open surgery, sigmoidectomy, a history of recent weight loss, and high APR-DRG mortality score (4) when compared to “early” readmission. Wick et al. similarly divided their cohort into “early” (post-discharge days 0–30) and “late” readmissions (post-discharge days 31–90), and only reported on factors associated with “early” readmissions, which included surgical site infection at index admission, primary diagnosis of colon cancer, proctectomy or colectomy, discharge disposition to non-home setting, index admission LOS ≥ 7 days, and APR-DRG severity of illness score 4 [9]. These findings suggest that with respect to emergency colectomies, sicker patients or more complicated procedures put patients at greater risk of having a complication that will require readmission in the “late” post-operative period.

The cost of readmission was slightly greater in the “late” readmission group [$37,936 (IQR $20,464–$73,835)] compared to the “early” group [$34,374 (IQR $18,386–$68,014] and this was also the case on linear regression, accounting for relevant covariates (OR $1717.09 USD; 95%CI $1717.05–$1717.12). While the difference in individual admissions may not be clinically relevant, the fact that more than half of the readmissions for patients who underwent emergency colectomies occur in a time period that is often overlooked by studies reporting on emergency colectomy (i.e., beyond POD30) makes the aforementioned findings impactful. Given the 11,524 patients readmitted in the “late” group, over $437 million would not have been captured for this cohort, which represents approximately $46 million per year over the 9 years represented in the study (half of each year captured).

This study supports the need for attention to longer follow-up post-emergency colectomy, especially since most preventable complications (e.g., stoma- and renal-related-complications) occurred in the “late” readmission group. Given the current state of most healthcare systems, expanding the follow-up for a sub-set of patients at greater risk of having a complication in the “late” post-operative period may be a more effective use of resources. This study demonstrated that patients with recent weight loss and those with higher post-operative risk scores for peri-operative morbidity and mortality were at increased risk of having “late” complications requiring readmission. With the recent adoption of the acute care surgery (ACS) models for emergency general surgery [10–12], emergency cases, including the emergency colectomies studied in this paper, are more often performed by on-call surgeons, as opposed to the sub-specialists who typically care for these patients on an elective basis. Anecdotally, the follow-up of patients operated on an emergency basis tends to be limited to the first post-operative visit usually coinciding with POD30, running the risk of missing “early” signs of symptoms that foreshadow preventable readmissions.

Major strengths of our study include cohort size and length of follow-up. The use of data from a large national dataset allows for logistic and linear regression analysis using multiple variables. Moreover, as data originated from all types of centers in the United States, representing patients from a wide variety of sociodemographic backgrounds, the observed findings are generalizable. This study was designed to limit information bias by having a standard follow-up time for all patients (6-month following emergency colectomy). This could also be interpreted as a limitation because it required reducing our cohort to half its original size, although there were no significant differences between our final cohort and the excluded cohort (data not shown). Furthermore, this could have also led to underestimating readmissions, as NRD only captures patients from the same state, meaning that if a patient moved to a different state following their colectomy, readmissions occurring in their new state would not be captured. Finally, the broad eligibility criteria and large size of this cohort also allowed for reduced selection bias in that all individuals originated from the same initial study population.

Several limitations of our study should be noted. First, using diagnostic and procedure codes for identifying post-operative complications does not precisely define colectomy-related complications and thus the list that we used was curated based on expert knowledge and an iterative review of the main diagnosis codes for readmitted patients. It is likely that there were relevant codes that were not used, and that the totality of colectomy-related complications may have been underestimated. Moreover, given the retrospective cohort design of this study, using a large national database, colectomy-related complications can only be associated with the pre-dating emergency colectomy and causation cannot be ensured. Use of another database, for example ACS-NSQIP, could have allowed this to be done, because as of 2012, a variable was included to indicate whether unplanned readmissions were related or unrelated to the index procedure; however, this database limits follow-up to 30-days post-discharge and thus could not be utilized for the long-term follow-up needed to perform the present study.

Second, the inability to review each patient’s chart prevented us from labeling a readmission as preventable or not, an important factor that has been studied in other smaller cohorts [4]. Despite stoma-related complications and dehydration being common reasons for readmission in our study, we could not judge whether these readmissions were indeed preventable. Finally, certain relevant covariates associated with post-operative complication risks (as demonstrated in the literature) such as race, ethnicity, frailty, and primary language spoken were not possible to assess using NRD [4, 13–15].

In summary, this study highlights that most complications after emergency colectomy likely occur outside of the POD30 window. Since there is no opportunity for pre-operative patient optimization due to the emergency nature of these colectomies, longer post-operative follow-up beyond 30 days is warranted. This will help to identify those at highest risk of readmission, and therefore, hopefully intervene before they require readmission or suffer secondary sequelae from the delay in addressing the complication. This will be beneficial for patient management, both medically and monetarily, thus helping reduce the burden on an already overstretched healthcare system.

### Supplementary Information

Below is the link to the electronic supplementary material.

Supplementary file1 (DOCX 15 kb)

Supplementary file2 (DOCX 31 kb)

Supplementary file3 (DOCX 19 kb)

Supplementary file4 (DOCX 20 kb)
