# Supplementary material for: Timing of readmissions for complications following emergency colectomy: follow-up beyond post-operative day 30 matters
Source: Surg Endosc. 2024 Mar 19;38(4):2240–51. doi: 10.1007/s00464-024-10724-y (PMC10978660; doi:10.1007/s00464-024-10724-y)
Supplement: Supplementary file 1 — Supplementary file1 (DOCX 15 kb) [file 464_2024_10724_MOESM1_ESM.docx]

**Appendix 1. Procedure codes used to define colectomy**

- ICD9 Codes
  - Lap colectomy
    - 17.32 (laparoscopic cecectomy)
    - 17.33 (laparoscopic right hemicolectomy)
    - 13.34 (laparoscopic resection of the transverse colon)
    - 17.35 (laparoscopic left hemicolectomy)
    - 17.36 (laparoscopic sigmoidectomy)
    - 17.39 (other laparoscopic partial excision of large intestine)
    - 45.81 (laparoscopic total intra-abdominal colectomy)
  - Open colectomies
    - 45.71 (open and other multiple segmental resection of large intestine)
    - 45.72 (open and other cecectomy)
    - 45.73 (open and other right hemicolectomy)
    - 45.74 (open and other resection of transverse colon)
    - 45.75 (open and other left hemicolectomy)
    - 45.76 (open and other sigmoidectomy)
    - 45.79 (other and unspecified partial excision of large intestine)
    - 45.82 (open total intra-abdominal colectomy)
- ICD10 Codes
  - Laparoscopic colectomies
    - 0DBE4ZZ (excision of large intestine, percutaneous endoscopic approach)
    - 0DTH4ZZ (excision of cecum, percutaneous endoscopic approach)
    - 0DTF4ZZ (excision of right intestine, percutaneous endoscopic approach)
    - 0DTL4ZZ (excision of transverse colon, percutaneous endoscopic approach)
    - 0DTG4ZZ (excision of left intestine, percutaneous endoscopic approach)
    - 0DTN4ZZ (excision of sigmoid colon, percutaneous endoscopic approach)
    - 0DTE4ZZ (excision of large intestine, percutaneous endoscopic approach)
    - 0DBE3ZZ (excision of large intestine, percutaneous approach)
    - 0DBE7ZZ (excision of large intestine, via natural or artificial orifice)
    - 0DBE8ZZ (excision of large intestine, via natural or artificial opening endoscopic)
    - 0DTH7ZZ (excision of cecum, via natural or artificial orifice)
    - 0DTH8ZZ (excision of cecum, via natural or artificial opening endoscopic)
    - 0DTF7ZZ (excision of right large intestine, via natural or artificial orifice)
    - 0DTF8ZZ (excision of right large intestine, via natural or artificial opening endoscopic)
    - 0DTL7ZZ (resection of transverse colon, via natural or artificial orifice)
    - 0DTL8ZZ (resection of transverse colon, via natural or artificial opening endoscopic)
    - 0DTLFZZ (resection of transverse colon, via natural or artificial opening with percutaneous endoscopic assistance)
    - 0DBLFZZ (excision of transverse colon, via natural or artificial opening with percutaneous endoscopic assistance)
    - 0DTG7ZZ (resection of left large intestine, via natural or artificial orifice)
    - 0DTG8ZZ (resection of left large intestine, via natural or artificial opening endoscopic)
    - 0DTGFZZ (resection of left large intestine, via natural or artificial opening with percutaneous endoscopic assistance)
    - 0DBGFZZ (excision of left large intestine, via natural or artificial opening with percutaneous endoscopic assistance)
    - 0DBMFZZ (excision of descending colon, via natural or artificial opening with percutaneous endoscopic assistance)
    - 0DTMFZZ (resection of descending colon, via natural or artificial opening with percutaneous endoscopic assistance)
    - 0DTN7ZZ (resection of sigmoid colon, via natural or artificial orifice)
    - 0DTN8ZZ (resection of sigmoid colon, via natural or artificial opening endoscopic)
    - 0DBNFZZ (excision of sigmoid colon, via natural or artificial opening with percutaneous endoscopic assistance)
    - 0DTNFZZ (resection of sigmoid colon, via natural or artificial opening with percutaneous endoscopic assistance)
    - 0DBE3ZZ (excision of large intestine, percutaneous approach)
    - 0DBE7ZZ (excision of large intestine, via natural or artificial opening)
    - 0DBE8ZZ (excision of large intestine, via natural or artificial opening with percutaneous endoscopic assistance)
  - Open colectomies
    - 0DBE0ZZ (excision of large intestine, open approach)
    - 0DTH0ZZ (resection of cecum, open approach)
    - 0DTF0ZZ (resection of right large intestine, open approach)
    - 0DTK0ZZ (resection of ascending colon, open approach)
    - 0DTL0ZZ (resection of transverse colon, open approach)
    - 0DTG0ZZ (resection of left large intestine, open approach)
    - 0DTN0ZZ (resection of sigmoid colon, open approach)
    - 0DBE0ZZ (excision of large intestine, open approach)
    - 0DTE0ZZ (resection of large intestine, open approach)
