# Supplementary material for: Timing of readmissions for complications following emergency colectomy: follow-up beyond post-operative day 30 matters
Source: Surg Endosc. 2024 Mar 19;38(4):2240–51. doi: 10.1007/s00464-024-10724-y (PMC10978660; doi:10.1007/s00464-024-10724-y)
Supplement: Supplementary file 3 — Supplementary file3 (DOCX 19 kb) [file 464_2024_10724_MOESM3_ESM.docx]

**Appendix 3. ICD 9/10 Diagnostic and Procedure Codes for Colectomy-Related Complications**

| **Category** | **Description** | **ICD-9-CM Codes** | **ICD-10-CM Codes** |
| --- | --- | --- | --- |
| Postoperative pain | Acute postoperative pain | 338.18 | G89.18 |
|  | Other chronic postoperative pain | 338.28 | G89.28 |
|  | Abdominal pain | 78900, 78909, 78900 | R109, R1010, R102, R1030 |
| Gastrointestinal | Persistent vomiting | 536.2, 564.3, 96.07, 78701 | K91.0, 0D9670Z, 0D9680Z, R11.10, R112 |
|  | Gastroparesis | 536.3 | K31.84 |
|  | Paralytic ileus | 560.1 | K56.0, K56.7 |
|  | Other and unspecified non-infectious gastroenteritis | 5589, 5559 | K529, K5289 |
|  | Toxic gastroenteritis and colitis | 5582 | K521 |
|  | Constipation | 56400 | K5900 |
|  | Acute vascular insufficiency of intestine | 5570 | K550 |
|  | TPN | 9915, 966 |  |
|  | Colonoscopy | 4525, 4523 | 0DJD8ZZ |
|  | Intestinal or peritoneal adhesions with obstruction | 560.81 | K56.5, K91.3 |
|  | Other or unspecified intestinal obstruction | 560.89, 560.9 | K56.69, K56.60, K56609, K56600 |
| Renal | Dehydration and volume depletion disorder | 276.51, 276.5 | E86.0, E86.9, E904.2 |
|  | Hypopotassemia | 2768 | E87.6 |
|  | Acute renal failure | 584, 584.5, 584.6, 584.7, 584.8, 584.9, 593.9 | N17.0, N17.1, N17.2, N17.8, N17.9 |
|  | Renal failure, not otherwise specified^*^ | 586 | N19 |
| Wound | Seroma complicating a procedure | 998.13 | T88.8XXA |
|  | Disruption of surgical wound | 998.30, 998.32 | T81.30XA, T81.31XA |
|  | Persistent postoperative fistula | 998.6 | T8183XA, T8183XD, T8183XS |
|  | Non-healing surgical wound | 998.83 | T81.89XA |
| Ventral/incisional hernia | Ventral hernia | 551.20, 551.29, 552.20, 552.29, 553.20, 553.29 | K43.6, K43.7, K43.9, K46.9 |
|  | Incisional hernia | 552.21, 553.21 | K43.0, K43.1, K43.2 |
| Anastomotic leak | Disruption of internal surgical wound | 998.31 | T81.82XA |
|  | Peritoneal abscess | 567.22 | K65.1 |
|  | Fistula of intestine | 569.81 | K63.2 |
|  | Other digestive system complications | 997.49 | K91.3, K91.8, K91.81, K91.82, K91.83, K91.89 |
| Bleeding | Hemorrhage complicating a procedure | 998.1, 998.11, 568.81 | K66.1, K91.61, K91.62, K91.840, K91.841, D78.01, D78.02, D78.21, D78.22, K76.01, L76.02, L76.21, L76.22 |
|  | Hematoma complicating a procedure | 998.12 | K91.61, K91.840 |
|  | Acute post-hemorrhagic anemia | 285.1 | D62 |
|  | Transfusion | 9904, 9907, 30233N1, 9905 |  |
|  | Anemia | 2859, 2809 | D649, D509 |
|  | Gastrointestinal bleeding | 5789, 5781, 5693 | K922, K921, K625 |
| Infection | Septicemia | 038.0, 038.1, 038.10, 038.11, 038.12, 038.19, 038.2, 038.3, 038.40, 038.41, 038.42, 038.43, 038.44, 038.49, 038.8, 038.9 | A40.9, A41.2, A41.01, A41.02, A41.1, A40.3, A41.4, A41.50, A41.3, A41.51, A41.52, A41.53, A41.59, A41.89, A41.9 |
|  | Septic shock | 785.52 | R65.21 |
|  | Shock without trauma | 785.59 | R57.1, R57.8 |
|  | Systemic inflammatory response syndrome due to infectious process^*^ | 995.91, 995.92 | A41.9, R65.20 |
|  | C difficil infection | 00845 | A047, A0471, A0472 |
|  | Aspiration pneumonia | 5070 | J690 |
|  | Infection and inflammatory reaction due to indwelling urinary catheter | 996.64 | T83.51XA |
|  | Postoperative shock | 998.00^*^, 998.02^*^, 998.09 | T81.10XA, T81.12XA, T81.19XA |
|  | Postoperative infected seroma, cellulitis, abscess | 998.51, 682.9, | T81.4XXA, L03.90, L03.91 |
|  | Postoperative infection, other | 998.5, 998.59 | T814XXA, T814XXD, T814XXS, K68.11 |
|  | Leukocytosis | 28860 | D72.829 |
| Venous thromboembolism (including PE) | Deep venous thromboembolism lower extremity | 451.81, 453.40, 453.41, 451.11, 451.19, 451.2, 453.42 | I82401, I82402, I82403, IB2409, I82411, I82411, I82412, I82413, I82419, I82421, I82422, I82423, I82429, I82431, I82432, I82433, I82439, I82442, I82443, I82449, I82491, I82492, I82493, I82499, I824Y1, I824Y2, I824Y3, I824Y9, I824Z1, I824Z2, I824Z3, I824Z9 |
|  | Other venous embolism and thrombosis | 451.9, 453.8, 453.9 | I80.9, I82.91 |
|  | Pulmonary Embolism | 415.11 | I26.99 |
| Interventions for post-operative complication | Re-laparotomy, Lavage | 54.12, 54.19, 54.25, 54.5, 54.51, 54.59 | 0WJG0ZZ, 3E1M38X, 3E1M38Z, 3E1H38X, 3E1H38Z, 3E1H88Z, 3E1G38X, 3E1G38Z, 3E1G78X, 3E1G78Z, |
|  | Percutaneous drainage | 54.91, 450.3, 481 | 0W9G30Z, 0W9J30Z, 0D9N30Z, 0D9P30Z, 0D9V30Z, 0W9G3ZX, 0W9G3ZZ, 0W9G40Z, 0W9G4ZX, 0W9G4ZZ, 0W9J3ZX, 0W9J3ZZ, 0W9J40Z, 0W9J4ZX, 0W9J4ZZ, 0D9W30Z, 0D9W3ZX, 0D9W3ZZ, 0D9W40Z, 0D9W4ZX, 0D9W4ZZ, 0D9E30Z, 0D9E3ZX, 0D9E3ZZ, 0D9E40Z, 0D9E4ZX, 0D9E4ZZ, 0D9E70Z, 0D9M30Z, 0D9M3ZX, 0D9M3ZZ, 0D9M40Z, 0D9M4ZX, 0D9M4ZZ, 0D9M70Z, 0D9M7ZX, 0D9M7ZZ, 0D9M80Z, 0D9M8ZX, 0D9M8ZZ, 0D9N30Z, 0D9N3ZX, 0D9N3ZZ, 0D9N40Z, 0D9N4ZX, 0D9N4ZZ, 0D9N70Z, 0D9N7ZX, 0D9P3ZX, 0D9P3ZZ, 0D9P40Z, 0D9P4ZX, 0D9P4ZZ, 0D9P70Z, 0D9P7ZX, 0D9S30Z, 0D9S3ZX, 0D9S3ZZ, 0D9S40Z, 0D9S4ZX, 0D9S4ZZ, 0D9V30Z, 0D9V3ZX, 0D9V3ZZ, 0D9V40Z, 0D9V4ZX, 0D9V4ZZ |
|  | Reclose postoperative disruption of abdominal wall | 54.61 | 0WQFXZZ |
|  | Lysis of adhesions | 5459 | 0DN80ZZ |
|  | Other incision with drainage of skin and subcutaneous tissue | 8604 |  |
| Stoma-related complications | Parastomal hernia, fistula, prolapse | 569.69, 569.89 | K43.3, K43.4, K43.5, K63.4, K94.19 |
|  | Infection of colostomy or enterostomy | 56961 | K94.02, K94.12 |
|  | Unspecified stoma complication | 569.60, 569.62, 569.69, E878.3, V55.2, V55.3 | K94.03, K94.09, K94.10, K94.13, K94.19Y83.3, Z433, Z432 |
| Failure to thrive | Other malaise and fatigue | 78079 | R53.1, K53.81, K53.83 |
|  | Physiotherapy | 9339 |  |
