# Supplementary material for: Timing of readmissions for complications following emergency colectomy: follow-up beyond post-operative day 30 matters
Source: Surg Endosc. 2024 Mar 19;38(4):2240–51. doi: 10.1007/s00464-024-10724-y (PMC10978660; doi:10.1007/s00464-024-10724-y)
Supplement: Supplementary file 4 — Supplementary file4 (DOCX 20 kb) [file 464_2024_10724_MOESM4_ESM.docx]

**Appendix 4. Characteristics of right-versus left-sided emergency colectomies**

| **Variable*** | **Right-sided colectomy (n=8,095)** | **Left-sided colectomy (9,522)** | **p-value** |
| --- | --- | --- | --- |
| Patient characteristics | | | |
| Age, years | 68 (55-78) | 65 (54-76) | <0.0001 |
| Sex  Female  Male | 4,469 (55.21%)  3,626 (44.79%) | 4,951 (52.00%)  4,571 (48.00%) | <.00001 |
| Area of origin  "Central" counties of metro areas of ≥1 million population  "Fringe" counties of metro areas of ≥1 million population  Counties in metro areas of 250,000-999,999 population  Counties in metro areas of 50,000-249,999 population  Micropolitan counties  Not metropolitan or micropolitan counties | 1,684 (27.62%)  1,548 (25.39%)  1,378 (22.6%)  596 (9.77%)  525 (8.62%)  366 (6.00%) | 1,932 (27.55%)  1,930 (27.53%)  1,567 (22.35%)  623 (8.88%)  549 (7.84%)  410 (5.85%) | 0.0505 |
| Income quartile  1  2  3  4 | 2,207 (27.66%)  2,018 (25.29%)  2,028 (25.42%)  1,726 (21.63%) | 2,562 (27.34%)  2,399 (25.59%)  2,417 (25.78%)  1,995 (21.28%) | 0.8505 |
| Comorbidities | | | |
| APDRG Severity of Illness Score  Minor loss of function**  Moderate loss of function  Major loss of function  Extreme loss of function | 824 (10.18%)  29 (29.2%)  38 (37.13%)  25 (23.47%) | 800 (8.4%)  2,769 (29.08%)  3,613 (37.94%)  2,339 (24.56%) | 0.0013 |
| APRDRG Risk of Mortality Score  Minor likelihood of dying  Moderate likelihood of dying  Major likelihood of dying  Extreme likelihood of dying | 1,805 (22.3%)  2,067 (25.53%)  2,711 (33.49%)  1,511 (18.67%) | 2,522 (26.49%)  2,379 (24.98%)  2,786 (29.26%)  1,834 (19.26%) | <0.0001 |
| AIDS | 7 (0.15%) | 12 (0.2%) | 0.4919 |
| Alcohol | 149 (2.99%) | 182 (2.94%) | 0.8993 |
| Deficiency anemia | 1,409 (28.26%) | 1,438 (23.28%) | <0.0001 |
| Arthritis | 141 (2.82%) | 242 (3.92%) | 0.0016 |
| Blood loss | 250 (5.02%) | 182 (2.94%) | <0.0001 |
| Congestive heart failure | 500 (10.03%) | 567 (9.17%) | 0.131 |
| Chronic lung disease | 914 (18.32%) | 1,152 (18.66%) | 0.6601 |
| Coagulopathy | 250 (5.02%) | 331 (5.37%) | 0.4127 |
| Depression | 471 (9.45%) | 623 (10.08%) | 0.2561 |
| Diabetes | 953 (19.11%) | 1,032 (16.71%) | 0.001 |
| Diabetes with complications | 183 (3.67%) | 158 (2.56%) | 0.0007 |
| Drug misuse | 104 (2.08%) | 143 (2.31%) | 0.4115 |
| Hypertension | 2,811 (56.37%) | 3,419 (55.37%) | 0.2868 |
| Hypothyroidism | 614 (12.31%) | 732 (11.86%) | 0.4584 |
| Liver disease | 121 (2.42%) | 160 (2.59%) | 0.5815 |
| Lymph nodes?? | 56 (1.12%) | 61 (0.99%) | 0.4855 |
| Electrolyte abnormalities | 2,010 (40.3%) | 2,608 (42.23%) | 0.0402 |
| Metastatic disease | 922 (18.49%) | 601 (9.73%) | <0.0001 |
| Neurologic disorder | 292 (5.86%) | 455 (7.37%) | 0.0015 |
| Obesity | 541 (10.84%) | 857 (13.88%) | <0.0001 |
| Para?? | 97 (1.95%) | 204 (3.3%) | <0.0001 |
| Peripheral vascular disease | 479 (9.61%) | 433 (7.02%) | <0.0001 |
| Psychiatric disease | 200 (4.01%) | 291 (4.72%) | 0.0724 |
| Pulmonary or circulatory disease | 172 (3.44%) | 164 (2.65%) | 0.0147 |
| Renal failure | 599 (12.01%) | 582 (9.42%) | <0.0001 |
| History of solid tumour | 236 (4.74%) | 211 (3.42%) | 0.0004 |
| Peptic ulcer disease | 4 (0.08%) | 4 (0.06%) | 0.7618 |
| Valve disease | 298 (5.97%) | 252 (4.09%) | <0.0001 |
| History of weight loss | 798 (16.00%) | 996 (16.13%) | 0.8577 |
| Disease characteristics | | | |
| Benign disease | 8,016 (99.02%) | 9,271 (97.36%) | <0.0001 |
| History of chemotherapy | 129 (1.59%) | 167 (1.75%) | 0.4095 |
| History of radiotherapy | 124 (1.53%) | 170 (1.79%) | 0.1905 |
| Colectomy characteristics | | | |
| Approach  MIS***  Open**** | 1,371 (16.94%)  6,724 (83.06%) | 1,539 (16.16%)  7,983 (83.84%) | 0.1681 |
| Index admission characteristics | | | |
| LOS, days | 10 (7-15) | 9 (7-14) | 0.0428 |
| Total charge, USD | 95,392 (58,543-161,712) | 90,596 (55,809-155,366) | 0.3398 |
| Disposition following emergency colectomy  Routine  Transfer to Short-term Hospital  Transfer Other*****  Home Health Care (HHC)  Against Medical Advice (AMA) | 3,816 (47.16%)  65 (0.8%)  2,305 (28.48%)  1,869 (23.1%)  37 (0.46%) | 3,225 (33.9%)  84 (0.88%)  2,947 (30.98%)  3,224 (33.89%)  33 (0.35%) | <0.0001 |
| Hospital type  Metropolitan non-teaching  Metropolitan teaching  Non-metropolitan | 2,766 (34.17%)  4,722 (58.33%)  607 (7.5%) | 3,609 (37.9%)  5,187 (54.48%)  726 (7.62%) | <0.0001 |
| Hospital size  Small  Medium  Large | 1,054 (13.02%)  2,184 (26.98%)  4,857 (60.00%) | 1,270 (13.34%)  2,769 (29.08%)  5,483 (57.58%) | 0.0031 |
| Population served by hospital  Large metropolitan areas with ≥1 million residents  Small metropolitan areas with <1 million residents  Micropolitan areas  Not metropolitan or micropolitan (non-urban residual) | 4,601 (56.84%)  2,887 (35.66%)  506 (6.25%)  101 (1.25%) | 5,547 (58.26%)  3,249 (34.12%)  584 (6.13%)  142 (1.49%) | 0.0898 |
| Origin vs hospital size concordance, *Yes* | 5,798 (95.1%) | 6,609 (94.27%) | 0.0352 |
